# Reproductive parameters of wild and hatchery‐reared sea trout ( *Salmo trutta* m. *trutta* L.) females from the Łeba River (southern Baltic Sea)

**DOI:** 10.1111/jfb.70283

**Published:** 2026-01-21

**Authors:** Adam M. Lejk, Piotr Hliwa

**Affiliations:** ^1^ Department of Logistics and Monitoring National Marine Fisheries Research Institute Gdynia Poland; ^2^ Department of Ichthyology and Aquaculture University of Warmia and Mazury in Olsztyn Olsztyn Poland

**Keywords:** anadromous brown trout, egg size, fish eggs, reproduction, salmonids

## Abstract

Sea trout *Salmo trutta* m. *trutta* L. has high socioeconomic and ecological importance in the Atlantic region; therefore, stocking hatchery‐reared fish is widely practiced for stock enhancement and recovery of wild populations. In this study, fecundity of wild and stocked hatchery‐reared sea trout sampled from the Łeba River (southern Baltic Sea basin) was evaluated. The average absolute fecundity (*F*_a_) was 5311 and 5338 eggs in the A.1+ age group, and 7897 and 9796 eggs in the A.2+ age group for wild and hatchery‐reared fish, respectively. Significant difference in the average *F*_a_ values between wild and hatchery‐reared female sea trout within the age groups was not observed. The relationships between *F*_a_ and fish length (FL) (log_10_ *F*_a_ = −1.6238 + 3.0544 × log_10_ FL, *R* ^2^ = 0.809) and between *F*_a_ and fish body weight (log_10_ *F*_a_ = 0.4845 + 0.9787 × log_10_ *W*_total_, *R* ^2^ = 0.874) were strongly positive. The relative fecundity of the analysed females was, on average, 2527 and 2652 eggs per 1 kg of total body weight for one‐ and two‐sea‐winter sea trout, respectively, and did not differ significantly regardless of sea age and fish origin. This study demonstrated that egg size was strongly related to fish size, but fish origin did not influence this relationship. Furthermore, no significant differences were found between the mean gonadosomatic index (GSI) values of hatchery‐reared and wild sea trout within the A.1+ and A.2+ age groups. Higher GSI values were observed, suggesting that Baltic sea trout invest more in gonad development than populations from other regions. Our results demonstrated that sea age only indirectly explained the absolute fecundity of sea trout, whereas the parameters that directly influenced fecundity were body length and weight. This study provides new insights for future stock recruitment analyses of Baltic sea trout and fills a gap in our knowledge of its reproductive parameters.

## 1 INTRODUCTION

Knowledge of fish fecundity is extremely important for the successful management and exploitation of fish stocks. Accurate assessment of fecundity is essential for stock recruitment analyses (Ricker, 1975). Furthermore, information on the individual fecundity of females is crucial when planning the number of spawners to be collected for stocking purposes (de Eyto et al., 2015). Salmonids have significant social and economic value in many countries around the world (Gillespie et al., 2018; ICES, 2025; O'Reilly & Mawle, 2007). Owing to ongoing climate change (Gallagher et al., 2022), it is necessary to deepen our knowledge of the basic biological aspects of fish species relevant to ecology and the economy.

The fish spawning season is a key stage for every population and determines its presence in the ecosystem (Bagenal, 1978). Individual fecundity indirectly affects fish population dynamics (Jonsson & Jonsson, 2017), and only the number of fertilised eggs ultimately determines the potential maximum number of new individuals in a population (Lobón‐Cerviá & Rincón, 2004). To maintain its position in the environment, each species reproduces to an extent that enables it to counteract all physical and biological hazards such as fisheries, food supply, predation and critical stages of its life‐history (Enberg et al., 2012). It is important to understand that environmental conditions can significantly modify fecundity and therefore affect production estimates. For instance, a higher food supply can boost growth rate, increase gonad weight, enhance fecundity at a given length and improve egg quality by increasing fat and yolk‐sac composition, resulting in enhanced embryonic development and hatching success (Jonsson & Jonsson, 1997; Kodela et al., 2023; Reading et al., 2018). Strong anthropogenic pressure negatively impacts aquatic ecosystems and generates ongoing changes in stream‐dwelling fish populations through their continuous adaptation (Belletti et al., 2020; Penczak, 2011).

Several studies have shown that egg production in salmonids differs according to fish size (Thorpe et al., 1984), growth rate (Quinn et al., 2004), age (Moffett et al., 2006) and spawning history (Reid & Chaput, 2012). Egg production varies among populations owing to life‐history strategies, morphs and physiological performance for environmental reasons (Jonsson & Jonsson, 1999; Morita & Takashima, 1998; Olofsson & Mosegaard, 1999). Variations in the time spent in freshwater and sea environments cause anadromous fish to grow to different sizes, resulting in variations in fecundity, as fecundity is largely determined by body size in many salmonid species (de Eyto et al., 2015; Domagała, 1986b; Reid & Chaput, 2012; Thorpe et al., 1984).

In fish species characterised by determinate fecundity, such as the anadromous brown trout *Salmo trutta* L. (sea trout), the number of oocytes is fixed at the beginning of the spawning period (Wootton & Smith, 2015). Furthermore, Jonsson and Jonsson (1999) reported a trade‐off between egg size and number within brown trout populations as reflected by the negative correlation between egg mass and fecundity adjusted for fish body size. For example, there is evidence that resident *S. trutta* living sympatrically with an anadromous morph produces fewer but larger eggs (Jonsson & Jonsson, 1999; Olofsson & Mosegaard, 1999). In general, trade‐offs between fecundity and egg size have been well documented for salmonids (e.g., Fleming & Gross, 1990; Jonsson et al., 1996; Smalås et al., 2017). This is a result of adaptation to site‐specific environmental conditions and variations between populations (e.g., Lobón‐Cerviá et al., 1997). In *S. trutta*, repeat spawners produced fewer but larger eggs than first‐time spawners of similar sizes. This pattern is observed in wild‐type and hatchery‐reared residents and anadromous trout (Jonsson & Jonsson, 1999). In *S. trutta*, the size of newly hatched fish strongly corresponds to egg size (Einum & Fleming, 1999; Elliott, 1984; Ojanguren et al., 1996). Moreover, offspring hatched from large eggs exhibit better growth and higher survival rates than those hatched from smaller eggs (Einum & Fleming, 1999). This is consistent with the results presented by Bagenal (1969) for resident *S. trutta*. He reported that better‐fed females produced more but smaller eggs than less‐well‐fed individuals. Now it is known that this phenomenon is regulated by environmentally induced reaction norms in optimal egg size, independent of genetic effects (Einum & Fleming, 1999; Olofsson & Mosegaard, 1999).

In fact, salmonid fishery management in many countries (e.g., France, England and Norway) is based on managing specific conservation limits, defined as the stock level that will achieve a long‐term sustainable yield (de Eyto et al., 2015). Stock enhancement and restoration programmes are conducted to support natural populations by producing and releasing stocking materials (Aprahamian et al., 2003; Hay & Hatton‐Ellis, 2006). Some studies have shown that hatchery‐reared fish grow differently from their wild counterparts (Jonsson et al., 1996; Jonsson & Jonsson, 2006; Lejk et al., 2021a). Because of the strong relationship between fish growth and fecundity (Bagenal, 1978), recruitment may depend on fish origin.

There are about a thousand populations of sea trout in the Baltic Sea basin, almost half of which reproduce and self‐sustain naturally in rivers (HELCOM, 2011). Moreover, sea trout have high socioeconomic and ecological importance in the Baltic Sea region. Both commercial and recreational catches have increased annually, which is a serious challenge for fishery managers (ICES, 2025). A previous literature review revealed gaps in our latest knowledge regarding fecundity parameters of sea trout populations from rivers flowing into the Baltic Sea and the amount of historical data used for other populations. To address this gap, extensive ovarian sampling was conducted to investigate the fecundity of wild and stocked hatchery‐reared sea trout during the spawning season. After 35 years, these are the first data on the fecundity parameters of individuals captured in a wild environment. Information on the size and condition of individual fish, as well as stock‐specific and biological data, was used to identify possible factors determining these variations in annual fecundity.

## 2 MATERIALS AND METHODS

### 2.1 Fish sampling

Using fyke nets, mature sea trout *Salmo trutta* m. *trutta* L. spawners were caught in Lake Łebsko (54°45′ N; 17°33′ E) during their spawning migration in November 2011–2015. Before analysis, the fish were euthanised using an overdose of MS‐222 (300 mg L^−3^; ACROS Organics, Belgium). After the cessation of vital reflexes, fish were transported under refrigerated conditions (approximately 4°C) to the laboratory, where the proper analysis was performed. Each fish was measured to determine fork length (FL, ±0.1 cm) and weight (*W*_total_, ±1 g). Because all hatchery‐reared smolts of Łeba River origin had been fin‐clipped before release into the lower section of the Łeba River, near its mouth, groups of fish originating from wild and farmed smolts could be distinguished. Spawners lacking an adipose fin were classified as the hatchery‐reared group (stocked as smolts at age 1+), whereas adult fish with an adipose fin were classified as the wild group originating from wild smolts. The latter group included individuals originating from both natural spawning and stocking with alevins released at the end of the yolk‐feeding phase. Both naturally spawned and stocked hatchlings exhibited a similar freshwater life history up to the smolt stage, as stocking occurred in a comparable period of natural emergence of fish from spawning redds (Radtke, 2013). Samples of approximately 15–20 scales were collected from each specimen for age determination (Elliott & Chambers, 1996). Then, whole ovaries were resected and weighed (*W*_ovary_) with an accuracy of ±1 mg using a MEDICAT 160 M electronic scale (Medicat Ltd., Switzerland). Individual somatic body weight *W*_som_ (±1 g) of analysed females was defined as the weight of an individual without gonads and stomach contents. The gonadosomatic index (GSI, %) was determined for individual females and calculated using the following formula (L'Abée‐Lund & Hindar, 1990):

$$\mathrm{GSI}=\frac{W_{\text{ovary}}}{W_{\mathrm{som}}}\times 100\%$$

where GSI is gonadosomatic index (%), *W*_ovary_ is ovary weight (g) and *W*_som_ is fish somatic weight (g).

The gravimetric method was used to determine the fecundity of individual fish (Murua et al., 2003). To calculate the absolute fecundity (*F*_a_), all oocytes (diameter >0.2 mm) were considered, assuming that those with a smaller diameter constitute the so‐called stock for the next spawning season. The relative individual fecundity (*F*_r_), that is, the number of eggs per unit of female total body weight, was calculated based on the individual total weight (*W*_total_, g) of the analysed individuals. Oocyte samples were collected from the central part of the ovary, weighed to the nearest 1 mg, and preserved in 3.6% buffered formalin solution (29.5 mM NaHPO_4_ × H_2_O and 460 mM Na_2_HPO_4_ × 2 H_2_O). After several days, the number of eggs in the collected subsamples (ovary fragments) was determined, and this procedure was repeated thrice. The obtained results were averaged and extrapolated to determine the total mass of eggs in the entire gonad to determine individual *F*_a_. The average weight of fish eggs (*W*_egg_, mg) was determined based on the number of eggs in the subsamples that were converted to subsample weight. The diameter of 10 oocytes collected from individual females (*n* = 40) was measured with an accuracy of ±1 μm using a LEICA MZ 16A stereoscopic microscope coupled to a computer, using the LEICA QWin Pro image analysis programme (LEICA Microsystems Ltd., Switzerland), following which the obtained values were averaged for each female (*D*_egg_, mm).

The relationships between *F*_a_ and different growth parameters were calculated based on the characteristics of the relationship expressed by the equation of the exponential function (Bagenal, 1978):

$$F_{a}=a\times x^{b}$$

where *F*_a_ is absolute fecundity, (*n* eggs), *x* is the fork length (FL), *l.caud*. (cm) or total body weight *W*_total_ (g). *a*, *b* are constants estimated based on empirical data.

Parameters *a* (intercept) and *b* (slope) of the linear regression model, along with their 95% confidence intervals (CI), were estimated using the least‐squares method. A logarithmic (log_10_) transformation was applied to the data to stabilise the variance and make the relationships more linear.

$$l{\mathrm{og}}_{10}F_{a}=\log_{10}a+b\times \log_{10}x$$

### 2.2 Statistical analysis

All statistical analyses were performed using Statistica 13.1 (Stat Soft, Inc., USA), with differences considered statistically significant at *p* < 0.05 (α = 0.05). Data were first tested for normality using the Shapiro–Wilk test (S‐W) and for homogeneity of variances using Levene's test. The effects of age group and origin (wild and hatchery‐reared) of sea trout on growth variables (FL, *W*_total_) and fecundity‐related parameters (*F*_a_, *F*_w_, *W*_egg_, *D*_egg_ and GSI) were evaluated using a two‐way analysis of variance (ANOVA). If the ANOVA showed a significant difference, Tukey's honest significant difference (HSD) post hoc test was used to identify which samples differed.

The relationships between growth variables and fecundity parameters of all sampled sea trout were tested using regression analysis. Moreover, for selected correlations determined by linear regression (log_10_‐transformed data), an analysis of covariance (ANCOVA) was used to find differences in slopes (*b*) or intercepts (*a*) (Zar, 2014). For the correlation between ovary and egg weights, a linear‐log model was used to show the right‐skewed distribution of *W*_ovary_ and the non‐linear relationship between these two variables.

Student's *t*‐test was also used to compare whether the calculated values of the *b* parameter differed significantly from the constant value of *b* = 3 for fish length and *b* = 1 for fish weight (Bagenal, 1978).

## 3 RESULTS

### 3.1 Fish characteristics

The river age of the analysed sea trout (*n* = 50) females remained constant in the stocked hatchery‐reared fish group (1‐year‐old smolts; *n* = 33), whereas among wild individuals, it ranged between 1‐year‐old (*n* = 8) and 2‐year‐old (*n* = 9) smolts. Mature females spend 1–3 years at sea. Because only one individual that spent 3 years (A.3+) in the sea was identified, in further analyses considering the sea age of the fish, only those individuals that spent 1 year (A.1+; *n* = 25) or 2 years (A.2+; *n* = 24) in the sea were considered. Due to the small sample size (*n* = 6), no separate analysis of fecundity parameters was performed for repeated spawners.

The average length of fish at age A.1+ was 57.4 cm and 55.9 cm for wild and hatchery‐reared sea trout, respectively. Similarly, the average lengths of wild and hatchery‐reared two‐sea winter females were 64.5 cm and 67.5 cm, respectively. These results were followed by the average body weight data that characterised one‐ and two‐sea‐winter females: 2138 g and 3106 g for wild fish, and 2038 g and 3703 g for hatchery‐origin fish, respectively. No significant differences in body length or weight were observed between wild and hatchery‐origin fish in either age group (Tukey's HSD post hoc test, *p* > 0.05).

### 3.2 Fecundity parameters

The individual *F*_a_ of the analysed females ranged from 3511 to 16,801 eggs per fish (Table 1). The lowest *F*_a_ value was recorded in an individual measuring 50.8 cm in length and weighing 1545 g, whereas the highest was observed in an individual measuring 73.0 cm and weighing 4968 g. The average *F*_a_ was 5311 and 5338 eggs, respectively, for wild and hatchery‐reared fish in the A.1+ age group, and 7897 and 9796 eggs, respectively, for wild and hatchery‐reared fish in the A.2+ age group (Table 1). There was no significant difference in the average *F*_a_ values between wild and hatchery‐reared female sea trout in the A.1+ age group (Tukey's HSD post hoc test, *p* = 0.999). Moreover, analysis showed that the mean values of *F*_a_ between wild and hatchery‐reared females in the A.2+ age group did not differ (Tukey's HSD post hoc test, *p* = 0.156). The available fish samples limited the possibility of conducting comparisons between all river age groups, but the comparison between 1‐year‐old and 2‐year‐old smolts (pooled sample of wild‐ and hatchery‐reared) showed no differences in fecundity within fish that spent one winter at sea (Tukey's HSD post hoc test, *p* = 0.908). Similarly, comparison of fecundity between 1‐year‐old and 2‐year‐old wild smolts showed no significant differences within one‐sea‐winter sea trout (Tukey's HSD post hoc test, *p* = 0.929). However, sea age significantly influenced *F*_a_ (two‐way ANOVA, *p* < 0.001).

**TABLE 1:** Absolute (*F*_a_) and relative (*F*_r_) fecundity of analysed sea trout females (*n* = 50); mean ± standard deviation (SD), range (minimum–maximum).

Group	Absolute fecundity, *F*_a_			Relative fecundity, *F*_r_			
	A.1+	A.2+	A.3+	A.1+	A.2+	A.3+	
Wild	5311 ± 1289.11^a^ (3606–8418)	7897 ± 1586.15^b^ (6152–9914)	‐	2527 ± 424.47^a^ (1766–3324)	2543 ± 178.44^a^ (2263–2773)	‐
Hatchery‐reared	5338 ± 1551.94^a^ (3511–9432)	9796 ± 2409.74^b^ (6220–16,801)	11,915†	2620 ± 324.12^a^ (2163–3031)	2652 ± 374.14^a^ (2078–3382)	2595†

*Note:* Values (within analysed parameter) marked with the same letter indexes did not show statistically significant differences (Tukey's HSD post hoc test, *p* > 0.05).

^†^ Only one individual aged A.3+ was identified in the analysed sample of fish.

The relative fecundity (*F*_r_) of the analysed sea trout females ranged from 1766 to 3382 eggs per 1 kg of fish total weight, with an average of 2527 and 2652 eggs for fish in age groups A.1+ and A.2+, respectively (Table 1). Moreover, relative fecundity did not differ regardless of sea age or fish origin (two‐way ANOVA, *p* > 0.05; Table 1).

### 3.3 Fecundity‐growth relationships

Analysis of females within specific age groups showed that the age of fish only indirectly influenced the *F*_a_ of sea trout (Table 1), whereas the parameters directly influencing fecundity were body length (*F*_[1,48]_ = 203.69, *p* < 0.001) and body weight (*F*_[1,48]_ = 331.79, *p* < 0.001) of individuals. A strong positive relationship was observed between *F*_a_ and fish length (log_10_ *F*_a_ = −1.6238 + 3.0544 × log_10_ FL, *R* ^2^ = 0.809) and between *F*_a_ and fish body weight (log_10_ *F*_a_ = 0.4845 + 0.9787 × log_10_ *W*_total_, *R* ^2^ = 0.874). The *b* coefficients of the regression equations did not differ significantly from 3 for body length (*t*‐test, *p* = 0.800) or from 1 for body weight (*t*‐test, *p* = 0.693). The calculated high values of the coefficient of determination *R* ^*2*^ and the correlation coefficient *r* indicated that both the body length (*R* ^2^ = 0.809) and body weight (*R* ^2^ = 0.874) of sea trout females were strongly correlated with fish fecundity, whereas for the relationship between *F*_a_ and female body weight, the *R* ^2^ value was higher.

Diagnostics of the estimated linear regression model (log_10_ *F*_a_ = 0.4845 + 0.9787 × log_10_ *W*_total_) for the relationship between *F*_a_ and body weight of sea trout females showed no discrepancies between the observed and modelled values of *F*_a_. There was no evidence to reject the assumption of normality in the distribution of residuals, as indicated by the Shapiro–Wilk test (*p* > 0.05).

**FIGURE 1:**
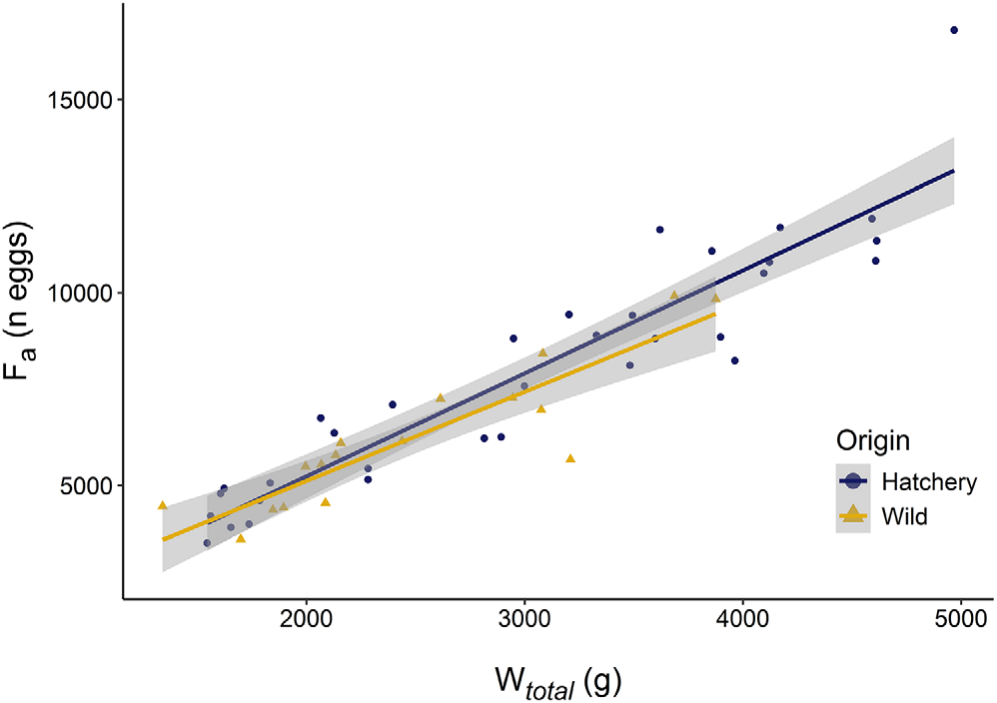
Relationship between absolute fecundity (*F*_a_) and total body weight (*W*_total_, g) of wild and hatchery‐reared sea trout females [regression line; wild: log_10_ *F*_a_ = 0.8189 + 0.8758 × log_10_ *W*_total_, *R* ^2^ = 0.773, df = 15, *p* < 0.001, ±95% confidence interval (CI); hatchery‐reared: log_10_ *F*_a_ = 0.452 + 0,99 × log_10_ *W*_total_, *R* ^2^ = 0.895, df = 31, *p* < 0.001, ±95% CI].

Separate analyses of the relationships between body weight and *F*_a_ for wild (*F*_[1,15]_ = 51.21, *p* < 0.001) and hatchery‐reared sea trout (*F*_[1,31]_ = 264.44, *p* < 0.001) revealed significant correlations between these two variables (Figure 1). However, this effect was similar between wild‐ and hatchery‐origin females (ANCOVA: comparison of slopes: *F*_[1,46]_ = 0.75, *p* = 0.390; comparison of intercepts: F_[1,47]_ = 1.35, *p* = 0.251). Moreover, an increase in fish body weight did not positively affect relative fecundity (F_[1,48]_ = 0.03, *p* = 0.866).

### 3.4 Eggs’ characteristics

The mean egg weight ranged from 56.02 to 90.95 mg and from 81.33 to 114.51 mg, for A.1+ and A.2+ age groups, respectively. There were no significant differences between the average egg weights of wild and hatchery‐reared females within the different age groups (Tukey's HSD post hoc test; A.1+: *p* = 0.969; A.2+: *p* = 0.998; Table 2). However, significant differences were observed in the average sea trout egg weights between the age groups (two‐way ANOVA, *p* < 0.001).

**TABLE 2:** Eggs characteristic of sampled sea trout from Łeba River (*n* = 50); mean ± standard deviation (SD), range (minimum–maximum).

Group	Egg weight *W*_egg_ (mg)			Egg diameter *D*_egg_ (mm)†		
	A.1+	A.2+	A.3+	A.1+	A.2+	A.3+
Wild	75.34 ± 12.03^a^ (56.02–90.95)	91.04 ± 6.01^b^ (81.33–96.71)	‐	4.99 ± 0.53^a^ (3.892–6.028)	5.36 ± 0.25^b^ (4.868–5.989)	‐
Hatchery‐reared	73.79 ± 6.63^a^ (64.04–88.08)	91.71 ± 7.85^b^ (82.28–114.51)	99.06‡	4.88 ± 0.31^a^ (3.791–5.519)	5.32 ± 0.28^b^ (4.523–6.204)	5.46‡

*Note*: Results (within analysed parameter) marked with the same letter indexes did not show statistically significant differences (Tukey's HSD post hoc test, *p* > 0.05).

^†^ Parameter determined on the basis of 40 females.

^‡^ Only one individual aged A.3+ was identified in the analysed sample of fish.

The diameter of sea trout eggs ranged from 3.79 to 6.20 mm, with an average [± standard deviation (SD)] of 5.18 ± 0.39 mm. The average values of egg diameter of fish aged A.1+ were 4.99 mm and 4.88 mm for wild and hatchery‐reared individuals, respectively (Table 2). Female sea trout that spent two winters at sea were characterised by larger eggs of an average diameter of 5.36 mm and 5.32 mm for wild and hatchery‐reared individuals, respectively. The egg diameter of one sea trout female classified at age A.3+ ranged from 5.02 to 5.71 mm, with an average (±SD) of 5.46 ± 0.21 mm (Table 2). No significant differences were observed between the average egg diameter values within the analysed age groups of wild and hatchery‐reared females (Tukey's HSD post hoc test; A.1+: *p* = 0.850; A.2+: *p* = 0.989), but differences were observed between one‐ and two‐sea winter fish (two‐way ANOVA, *p* < 0.001).

Egg weight (*F*_[1,48]_ = 60.32; *p* < 0.001) was significantly related to fish body weight. Linear regression analysis between body weight and egg weight of wild (log_10_ *W*_egg_ = 0.7130 + 0.3521 × log_10_ *W*_total_, *R* ^2^ = 0.362) and hatchery‐reared (log_10_ *W*_egg_ = 0.8691 + 0.3050 × log_10_ *W*_total_, *R* ^2^ = 0.692) sea trout revealed significant correlations between these two parameters. However, no differences were found between the wild and hatchery‐reared fish (ANCOVA: comparison of slopes: *F*_[1,46]_ = 0.22, *p* = 0.642; comparison of intercepts: *F*_[1,47]_ = 0.08, *p* = 0.779).

**FIGURE 2:**
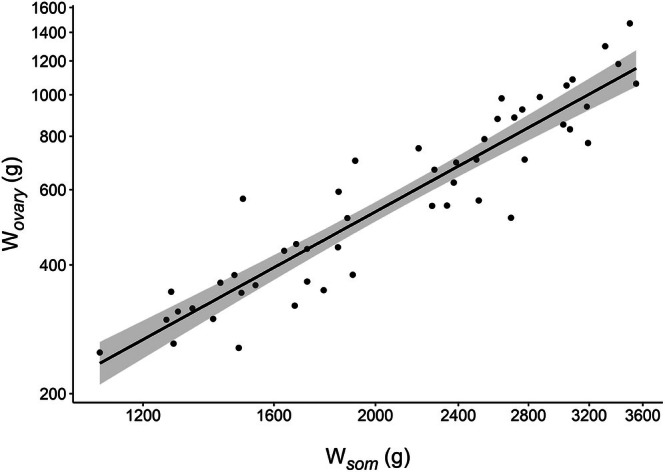
Relationship between somatic weight (*W*_som_, g) and ovary weight (*W*_ovary_, g) of sea trout females [pooled sample; regression line: log_10_ *W*_ovary_ = −1.7205 + 1.3472 × log_10_ *W*_som_ ± 95% confidence interval (CI), *R* ^2^ = 0.861, df = 48, *p* < 0.001].

**FIGURE 3:**
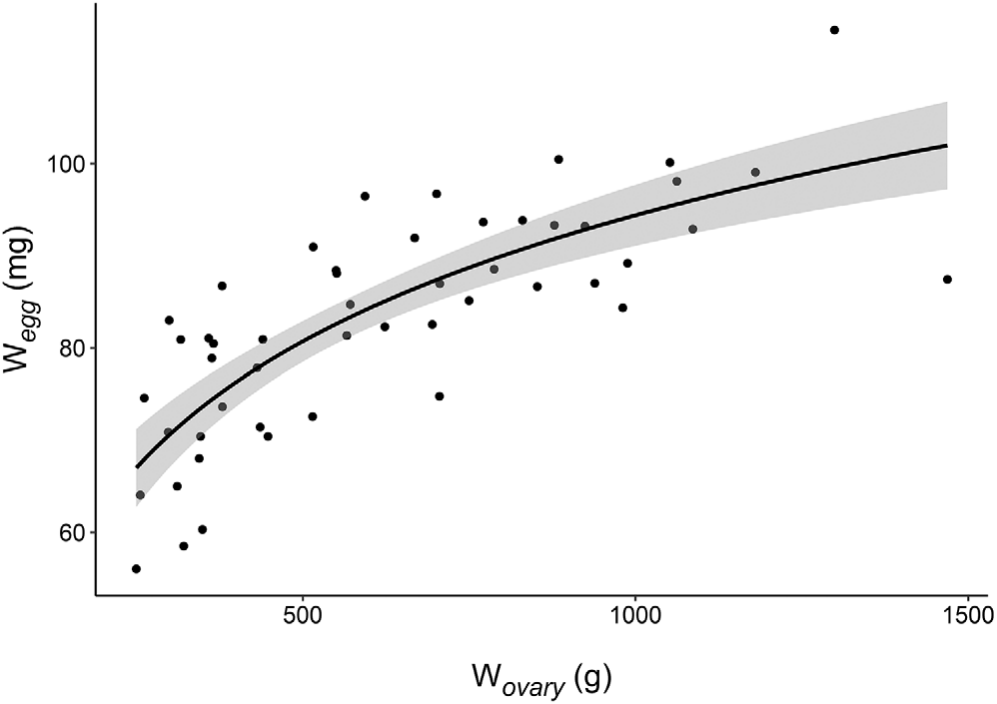
Relationship between ovary weight (*W*_ovary_, g) and mean egg weight (*W*_ooc_, mg) of sea trout females [pooled sample; regression line: *W*_egg_ = −42.0279 + 45.4732 × log_10_ *W*_ovary_ ± 95% confidence interval (CI), *R* ^2^ = 0.623, df = 48, *p* < 0.001].

Total ovary weight increased with fish somatic weight (*F*_[1,48]_ = 297.97, *p* < 0.001; Figure 2). There was a linear relationship between the somatic weight and ovary weight of the analysed sea trout (pooled sample: log_10_ *W*_ovary_ = −1.7206 + 1.3472 × log_10_ *W*_som_, *R* ^*2*^ = 0.861). This pattern was similar for wild and hatchery‐reared females (ANCOVA: comparison of slopes: *F*_[1,46]_ = 0.63, *p* = 0.433; comparison of intercepts: *F*_[1,47]_ = 1.29, *p* = 0.262). Furthermore, the ovary weight consequently influenced the average egg weight (*F*_[1,48]_ = 79.15, *p* < 0.001; Figure 3), but this effect was observed to be stronger in small females and levelled off in bigger individuals (≥3.5 kg) characterised by large gonads.

### 3.5 Gonadosomatic index

**FIGURE 4:**
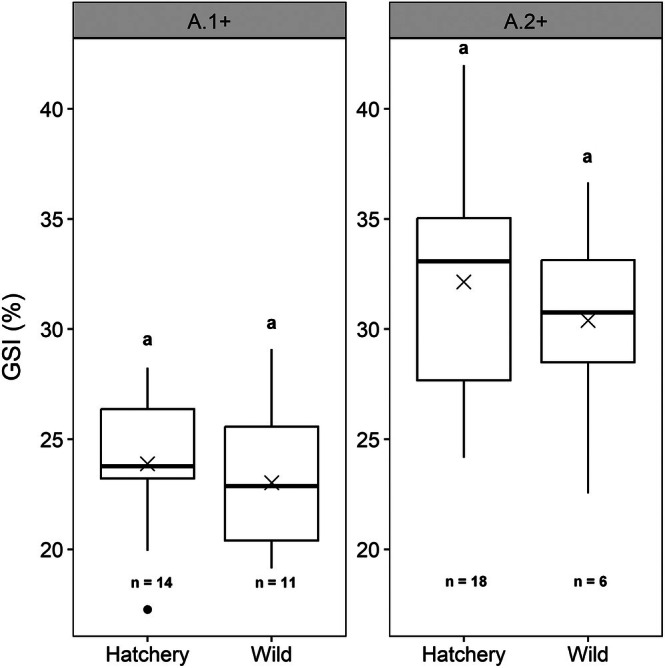
Gonadosomatic index (GSI, %) of wild and hatchery‐reared sea trout females (*n* = 49) in A.1+ and A.2+ age groups; lines, crosses, boxes and whiskers represent medians, means, the 25th and 75th percentiles and minimum and maximum values, respectively. Black points indicate outlier values. Results within each age group marked with the same letter indexes did not differ significantly (Tukey's HSD post hoc test, *p* > 0.05).

No significant differences were observed between the mean values of the gonadosomatic index (GSI) of sea trout females originating from stocking with smolts (GSI = 23.88 ± 3.09, *n* = 14) and wild fish (GSI = 23.03 ± 3.30, *n* = 11) within the A.1+ age group (Tukey's HSD post hoc test, *p* = 0.960; Figure 4). Furthermore, no differences were observed between the mean values of the GSI of sea trout females originating from stocking with smolts (GSI = 32.15 ± 5.31, *n* = 18) and wild fish (GSI = 30.40 ± 4.88, *n* = 6) within the A.2+ age group (Tukey's HSD post hoc test, *p* = 0.824; Figure 4). However, the difference in the mean values of the GSI between the two age groups of the examined wild and hatchery‐reared females was statistically significant (two‐way ANOVA, *p* < 0.001).

## 4 DISCUSSION

We evaluated selected fecundity parameters of wild and stocked hatchery‐reared sea trout females, caught from the Łeba River, a small river that flows into the southern Baltic Sea. The estimated absolute fecundity values were within the ranges reported in the existing literature for anadromous populations of *Salmo trutta* L. from the southern Baltic Sea region (Domagała, 1986a, 1986b; Morawska, 1967) as well as from other regions such as the British Isles, Norway and France (Jonsson & Jonsson, 1999; Solomon, 1997).

In the present study, the river age was unchanged in the hatchery‐reared fish group (1‐year‐old smolts), whereas in wild individuals, it varied between 1‐ and 2‐year‐old smolts. No differences in fecundity were observed in a group of one‐sea‐winter wild fish, suggesting that a similar pattern might occur in fish that spent 2 years at sea. Unfortunately, the number of available fish samples was not sufficiently large to validate this hypothesis. It should be noted that the presented analysis is exploratory and that the conclusions are highly tentative. We confirmed that the age of the fish at sea only indirectly explained the absolute fecundity of sea trout, whereas the parameters directly influencing fecundity were body length and weight. In our study, the difference in mean fecundity between age groups A.1+ and A.2+ was approximately 3500 eggs. A similar conclusion was presented by Morawska (1967) for sea trout taken from the Vistula River. Morawska (1967) reported that the time spent in the river did not affect female fecundity. In contrast, Domagała (1986b) reported that female sea trout that migrated to the sea after their first year in the river were characterised by higher fecundity than individuals that underwent smoltification after 2–3 years, with a correspondingly lower average number of eggs per kilogram of body weight. Similar patterns were reported by Thorpe et al. (1984) for Atlantic salmon *Salmo salar* L. across the studied river age groups; fecundity showed a uniform increase with female length; however, at any given length, fish that spent either one or two winters in the river had significantly greater fecundity than those that spent three winters before smoltification. They also noticed that sea age was responsible for some of the variation in egg number. This was partly confirmed by de Eyto et al. (2015), who found that neither river nor sea age was a source of variation in Atlantic salmon fecundity.

The present results show a similar relationship between fish absolute fecundity and total length or body weight, as reported in other studies on anadromous (Elliott, 1995; L'Abée‐Lund & Hindar, 1990), resident (Jonsson & Jonsson, 1999; Lobón‐Cerviá et al., 1997) and lacustrine (Sakowicz, 1961) *S. trutta* populations. This pattern has also been observed in different salmonids (Jonsson et al., 1996; Loewen et al., 2010; Morita & Takashima, 1998; Quinn & Bloomberg, 1992) and other fish species such as *Vimba vimba* L. (Hliwa & Martyniak, 2002). In contrast, fish growth did not positively affect relative fecundity, resulting in similar values, regardless of fish origin. Furthermore, we observed that ovary weight increased with fish somatic weight, and this relationship was similar for wild and hatchery‐reared sea trout. Jonsson et al. (1996) came to a similar conclusion when examining Atlantic salmon; however, they also suggested that the growth rate in either freshwater or at sea did not improve the explanatory power of the relationship.

Egg size positively correlated with fish size. This is consistent with results reported for *S. trutta* (e.g., Chełkowski et al., 1990; Jonsson & Jonsson, 1999; Lobón‐Cerviá et al., 1997) and *S. salar* (e.g., Thorpe et al., 1984; Jonsson & Jonsson, 1996; Moffett et al., 2006). Furthermore, we found that older and, therefore, larger two‐sea winter sea trout females produced approximately 20% larger ova (in diameter and weight) than one‐sea winter females. This is relevant to reproductive success, causing the size of newly hatched fish to be positively correlated with egg size (Elliott, 1984; Ojanguren et al., 1996; Thorpe et al., 1984) and consequently may increase salmonid alevin and fry survival (Einum & Fleming, 1999). In contrast, Moffett et al. (2006) reported that the river age of Atlantic salmon females explained the majority of the variation in egg diameter and suggested that larger eggs from 2.1+ adults produced longer and heavier swim‐up fry than smaller eggs from 1.1+ adults. Moreover, they suggested that the survival of eggs from 1.1+ adults was lower than that of eggs from 2.1+ adults during the swim‐up stage. This finding led us to assume that the same pattern may be observed in sea trout, especially because some authors have found an influence of river age on egg production. The observed effect of the curved relationship between ovary weight and egg weight was similar to that reported for Atlantic salmon, in which the somatic weight of females was used as a predictor (de Eyto et al., 2015).

Gonadal investment, as expressed by the GSI, increased with sea age of female sea trout but did not differ based on fish origin. Studies on salmonids commonly report female GSIs of approximately 20%–25% (Fleming, 1996; Jonsson & Jonsson, 1997; L'Abée‐Lund & Hindar, 1990). Our results showed higher GSI values, suggesting that Baltic sea trout invest more in gonad development than populations from other regions. This trend was particularly noticeable among older females. Sampling time and fish maturity stages reported by previous studies (Jonsson & Jonsson, 1997; L'Abée‐Lund & Hindar, 1990) are perfectly aligned with the current study. They also used somatic fish weight to calculate GSI. These arguments demonstrate the validity of making comparisons. Higher GSI values may be the result of regional adaptations to the specific environmental conditions of rivers flowing into the southern Baltic Sea and may reflect the species' ecological plasticity. Jonsson and Jonsson (1997) reported that gonadal investment was higher for anadromous than for resident *S. trutta* in southern Norway. They found that sea‐run females were characterised by a GSI of 21.5%, whereas resident individuals had a GSI of 15.8%. Similar results have been reported in anadromous brown trout populations in western Norway (L'Abée‐Lund & Hindar, 1990). In general, salmonid females invest a higher proportion of their weight in gonads than males, which is explained by the fact that the reproductive success of females is more dependent on gonadal production, whereas in males, it is less important (Fleming, 1996; Jonsson & Jonsson, 1997).

The results of the present study showed that body weight has a significant effect on absolute fecundity in female trout. They also confirmed the hypothesis that body weight positively affects egg size. However, in the tested fish samples, there were no differences in fecundity or other reproductive parameters between wild and hatchery‐reared individuals. It should be noted, however, that the sample size particularly for the wild fish group was limited. This constitutes a significant limitation of the study and may have reduced the statistical power of the analyses. As a result, the lack of statistically significant differences between wild and hatchery‐reared individuals should be interpreted with caution. A non‐significant result does not necessarily imply the absence of a true biological difference; it may instead reflect insufficient statistical power to detect such a difference. Therefore, the conclusion regarding the lack of effect of fish origin on reproductive traits should be considered preliminary. This is also supported by the lack of differences in the body weight of the sampled females depending on their origin, which was shown in a much larger sample, as reflected by the significantly lower body weight of hatchery‐reared females at age A.1+ (average body weight: wild: 1984 g, *n* = 191; hatchery‐reared: 1717 g, *n* = 481; Lejk et al., 2021b). The observed differences in body weights of wild and hatchery‐reared females may affect fecundity and reproductive success, particularly in individuals migrating to spawn after their first winter at sea. This may have resulted in differences in the initial length and weight of hatched larvae between wild‐ and hatchery‐origin females. This is because larger females produce larger eggs (Brooks et al., 1997; Chełkowski et al., 1985; Jonsson & Jonsson, 1999), which consequently produce larger larvae (Kamler, 1992). This results in better subsequent growth and higher offspring survival rates (Bagenal, 1969). Depending on the proportion of females of wild and hatchery origin in a given spawning season, there may be an increase or decrease in the frequency of particular cohorts in a given population. Unfortunately, the limited number of repeating spawners makes it impossible to conduct a separate reliable fecundity analysis, even though their share in the spawning stock is estimated to be 9.0% for second‐time spawners and 0.8% for third‐time spawners (Lejk et al., 2021b). This indicates that repeat spawners are currently not a significant component of the sea trout population from the Łeba River. An important addition to current knowledge would be the analysis of the quality of semen from wild and farmed male sea trout from the Łeba River. The results of a similar study indicate reduced concentration, mortality and vitality of sperm from farmed fish (Skjæraasen et al., 2009).

The present study provides fecundity estimates for a sea trout population from a river flowing into the southern Baltic Sea. The presented data provide new insights for future stock‐recruitment analysis of the unique Baltic sea trout and can also be applied to other populations. Notably, differences in fecundity‐related relationships between sea trout populations have been reported in the literature; therefore, studies regarding this should strive to collect river‐specific data (Elliott, 1995; L'Abée‐Lund & Hindar, 1990). Moreover, various studies have shown large variations in fish size between years within a given salmonid population, as well as between populations, which may cause stock recruitment estimates to be inaccurate. The strong dependence of fecundity on fish size highlights the need to collect accurate data on the population size structure of each river (Elliott, 1995; King et al., 2023; Moffett et al., 2006). The results of the present study may contribute significantly to the work of the ICES Working Group to Develop and Test Assessment Methods for Sea Trout Populations (WGTRUTTA), which develops and evaluates different ways to model sea trout populations and brings together scientists from across the native range of sea trout (ICES, 2024). Moreover, this crucial knowledge has a wide range of applications and can be used in stock enhancement programmes. In comparison to previously published works, the present study takes into account wild and hatchery‐reared (stocked) sea trout, which are of great importance in situations of intensive stocking and for conservation purposes.

## AUTHOR CONTRIBUTIONS

Conceptualisation: A.M.L. and P.H. Data curation: A.M.L. Formal analysis: A.M.L. and P.H. Funding acquisition: A.M.L. Investigation: A.M.L. and P.H. Methodology: A.M.L. and P.H. Project administration: A.M.L. Visualisation: A.M.L. Writing – original draft: A.M.L. and P.H.

## FUNDING INFORMATION

This work was funded by a grant from the National Science Centre, Poland, PRELUDIUM 4 (contract DEC‐2012/07/N/NZ9/01321). Part of this work was co‐financed by statutory project nos. 08020801 and GW/2011/15 conducted at the Department of Fish Biology and Pisciculture, University of Warmia and Mazury in Olsztyn.

## CONFLICT OF INTEREST STATEMENT

The authors have no conflicts of interest to declare.

## Data Availability

The data that support the findings of this study are available from the corresponding author upon reasonable request.
